# P-1456. Re-evaluating Economic & Health Impact of PCV20 in Adults Using Real-world Effectiveness Data

**DOI:** 10.1093/ofid/ofaf695.1642

**Published:** 2026-01-11

**Authors:** Jeffrey T Vietri, Euan Dawson, Michael Bois, Paula Peyrani, Jelena Vojicic, Mark Rozenbaum

**Affiliations:** Pfizer, Inc., Collegeville, Pennsylvania; Pfizer Inc, Walton Oaks, England, United Kingdom; Pfizer, New York, New York; Pfizer, Inc, Collegeville, PA; Pfizer Canada, Kirkland, QC, Canada; Pfizer Inc., Randstad, Noord-Holland, Netherlands

## Abstract

**Background:**

Invasive pneumococcal disease (IPD) and community-acquired pneumonia pose significant health risks, particularly for older adults and those with underlying conditions. In many countries including the United States (US), vaccine policy and reimbursement decisions are informed by cost-effectiveness analysis (CEA). However, CEA is sensitive to key inputs—such as vaccine effectiveness (VE)—uncertain at time of initial review but more certain after widespread use of the vaccine. This study incorporates recently presented real-world VE of 20-valent pneumococcal conjugate vaccine (PCV20) into an established cost-effectiveness model.

**Methods:**

A published cost-effectiveness model was adapted to current US epidemiology and estimated lifetime impact of adult PCV20 use on IPD from any serotype, all-cause pneumonia, deaths, quality-adjusted life-years (QALYs), and costs on single-year cohorts of vaccine-naïve US adults using 1) VE inputs used by the Centers for Disease Control and Prevention (CDC) and 2) VE calculated from real-world data on PCV20 effectiveness against IPD and all-cause pneumonia in Medicare beneficiaries aged ≥65 years. Other model inputs (e.g., disease incidence, serotype distribution, associated costs) were taken from recent presentations of the CDC/Tulane pneumococcal cost-effectiveness model to reflect typical evaluation of adult pneumococcal vaccines.

**Results:**

Relative to use of CDC VE assumptions, use of real-world VE inputs predicted 234 more IPD cases but 6,960 fewer inpatient pneumonia cases, 31,313 fewer outpatient cases and 166 fewer deaths, yielding 1,995 more QALYs and offsetting $332M more in medical costs over the lifetime of a cohort of 50 year old adults. The difference between the two methods was larger in a single cohort of adults aged 65 years at vaccination, with 134 more IPD cases but 35,358 fewer inpatient pneumonia cases, 78,940 fewer outpatient cases, and 1,258 fewer deaths, yielding 8,492 QALYs and offsetting $736M more in medical costs over the remaining lifetime of the cohort.

**Conclusion:**

Use of real-world VE reflected much greater public health and economic benefits of adult PCV20 use than using VE previously assumed. Policymakers should consider emerging real-world PCV20 VE data when considering adult pneumococcal recommendations.

**Disclosures:**

Jeffrey T. Vietri, PhD, Pfizer Inc: Employment|Pfizer Inc: Stocks/Bonds (Public Company) Euan Dawson, MSc, Pfizer Inc: Employment Michael Bois, PhD, M(ASCP), PFIZER: Stocks/Bonds (Public Company) Paula Peyrani, MD, Pfizer, Inc: Employee|Pfizer, Inc: Stocks/Bonds (Public Company) Jelena Vojicic, MD, Pfizer Inc.: Employee|Pfizer Inc.: Stocks/Bonds (Public Company)|Pfizer Inc.: Stocks/Bonds (Public Company) Mark Rozenbaum, PhD, M.B.A., Pfizer: Stocks/Bonds (Public Company)

